# Repurposed quinacrine synergizes with cisplatin, reducing the effective dose required for treatment of head and neck squamous cell carcinoma

**DOI:** 10.18632/oncotarget.27156

**Published:** 2019-08-27

**Authors:** Jennifer Bryant, Nikolaos Batis, Anna Clara Franke, Gabriella Clancey, Margaret Hartley, Gordon Ryan, Jill Brooks, Andrew D. Southam, Nicholas Barnes, Joanna Parish, Sally Roberts, Farhat Khanim, Rachel Spruce, Hisham Mehanna

**Affiliations:** ^1^Institute of Head and Neck Studies and Education (InHANSE), University of Birmingham, Birmingham, UK; ^2^Institute of Cancer and Genomic Sciences, College of Medical and Dental Sciences, University of Birmingham, Birmingham, UK; ^3^University Hospitals Birmingham NHS Foundation Trust, Birmingham, UK; ^4^School of Biosciences, University of Birmingham, Birmingham, UK; ^5^Phenome Centre Birmingham, University of Birmingham, Birmingham, UK; ^6^School of Biomedical Sciences, Institute of Clinical Sciences, University of Birmingham, Birmingham, UK; ^7^School of Pharmacy, Institute of Clinical Sciences, College of Medical and Dental Sciences, University of Birmingham, Birmingham, UK; ^*^Joint first authors; ^#^Joint senior authors

**Keywords:** head and neck cancer, drug repurposing, drug repositioning, quinacrine, mepacrine

## Abstract

Despite highly toxic treatments, head and neck squamous cell carcinoma (HNSCC) have poor outcomes. There is an unmet need for more effective, less toxic therapies. Repurposing of clinically-approved drugs, with known safety profiles, may provide a time- and cost-effective approach to address this need.

We have developed the AcceleraTED platform to repurpose drugs for HNSCC treatment; using *in vitro* assays (cell viability, clonogenic survival, apoptosis) and *in vivo* models (xenograft tumors in NOD/SCID/gamma mice).

Screening a library of clinically-approved drugs identified the anti-malarial agent quinacrine as a candidate, which significantly reduced viability in a concentration dependent manner in five HNSCC cell lines (IC50 0.63–1.85 μM) and in six primary HNSCC samples (IC50 ~2 μM). Decreased clonogenic survival, increased apoptosis and accumulation of LC3-II (indicating altered autophagy) were also observed. Effects were additional to those resulting from standard treatments (cisplatin +/– irradiation) alone. *In vivo*, daily treatment with 100 mg/kg oral quinacrine plus cisplatin significantly inhibited tumor outgrowth, extending median time to reach maximum tumor volume from 20 to 32 days (*p* < 0.0001) versus control, and from 28 to 32 days versus 2 mg/kg cisplatin alone. Importantly, combination therapy enabled the dose of cisplatin to be halved to 1 mg/kg, whilst maintaining the same impairment of tumor growth. Treatment was well tolerated; murine plasma levels reached a steady concentration of 0.5 μg/mL, comparable to levels achievable and tolerated in humans.

Consequently, due to its favorable toxicity profile and proven safety, quinacrine may be particularly useful in reducing cisplatin dose, especially in frail and older patients; warranting a clinical trial.

## INTRODUCTION

Head and neck squamous cell carcinoma (HNSCC) is a debilitating disease comprising 600,000 cases per year worldwide [1]. For advanced stage disease, treatment consists primarily of either chemoradiotherapy or surgical intervention with adjuvant treatment, but results in a poor five year survival rate - around only 50% [2]. Due to the high morbidity of current treatments, quality of life is severely impaired, with evident unmet need necessitating more effective therapies with lower toxicity; especially as recent studies examining cetuximab as a less toxic alternative for low risk human papillomavirus positive HNSCC have shown similar toxicity, and lower efficacy compared to cisplatin [3, 4].

Repurposing existing drugs for cancer therapy can be valuable due to known safety profiles leading to higher success rates and reduced development times, and subsequently lower costs compared with the development of novel therapeutics. The time taken to gain clinical approval for repurposed drugs is usually considerably shorter (3–12 years vs 10–17 years for novel therapeutics) [5], and success rates for market approval approach nearly 30% compared to only around 10% for new drugs [6].

We established a multi-stage drug discovery and repurposing platform called AcceleraTED. On initial screening against HNSCC cell lines, quinacrine was identified as a potential hit. Quinacrine, also known as mepacrine, was initially utilized as an antimalarial agent as early as the 1930s and is considered safe, with only minimal side effects, such as headaches and gastrointestinal upset [7]. Quinacrine has also been proposed as a treatment for numerous other indications, from colitis [8] to prion disease [9]. Vassey et al. (1955) [10] first assessed quinacrine as an anticancer agent in mice bearing several types of tumor, such as fibrosarcoma and carcinoma. More recently, effectiveness has been demonstrated in endometrial [11], colon [12], non-small cell lung [13] and HNSCC [14].

The mechanisms through which quinacrine exerts its anticancer effects are not fully understood. Quinacrine is a potent late-stage autophagy inhibitor [15] and has been shown to prime cells to the effects of cisplatin via apoptosis in cervical and endometrial cancer [16, 17]. Further mechanistic insights have been demonstrated in HNSCC whereby quinacrine was able to restore the function of the tumor suppressive protein, tumor protein 53 (TP53), leading to enhanced capabilities of initiating apoptotic cell death following DNA damage with cisplatin chemotherapy [14]; Moreover, quinacrine treatment has been shown to suppress phosphoinositide 3-kinase (PI3K), protein kinase B (AKT), mechanistic target of rapamycin (mTOR) and nuclear factor kappa-light-chain-enhancer of activated B cells (NF-κB) pathways [18].

Due to promising results from high throughput screening and the supportive literature, we sought to advance the repurposing potential of quinacrine as an anti-cancer therapy for HNSCC.

## RESULTS

### Quinacrine reduces cell viability of HNSCC cancer cell lines

**Figure 1:**
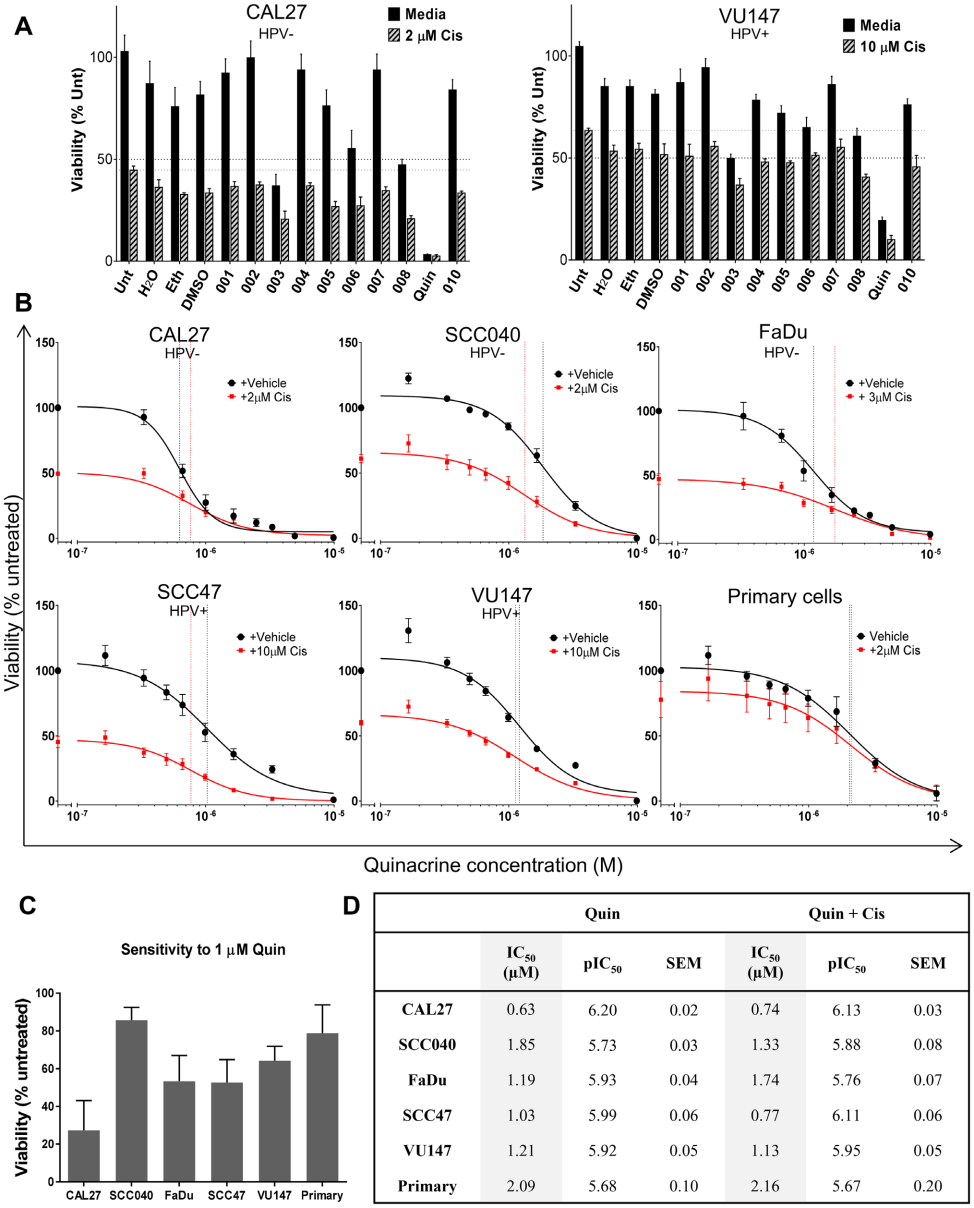
(**A**) Confirmatory screen for hits using CAL27 and VU147 cells exposed to various repurposed drugs at C_max_ (or lower), with and without cisplatin (*n* = 3). (**B**) Cell viability is compared to untreated controls following 72 (cell lines *n* = 3–7) or 96 (patient-derived primary tumor cells *n* = 6) hours exposure to quinacrine alone (black line) at increasing concentrations and also in the presence of cisplatin (red line). IC_50_ values are highlighted by vertical dotted lines color matched; responses fitted to a five-parameter logistic equation. (**C**) Viability of cells exposed to 1 μM quinacrine compared to untreated controls. (**D**) Summary of IC_50_ values (shaded columns) and inverse log of the IC_50_ values (pIC_50_) of quinacrine and standard error of the mean (SEM) in each cell line, with and without the addition of cisplatin.

The Prestwick library of 1280 FDA-approved drugs was initially screened at 10 μM using a high-throughput microplate platform. We identified the potential hits and completed a confirmatory screen in CAL27 and VU147 cells (Figure 1A), that revealed the ability of the anti-malarial drug, quinacrine, tested at 3.3 μM, to reduce cell viability by 96.8% and 80.7% in CAL27 and VU147 cells, respectively, compared to controls. Even higher reductions in cell viability were achieved when using quinacrine in combination with 2 μM cisplatin, causing 97.4% and 90.0% reduction in viability in CAL27 and VU147 cell lines, respectively (Figure 1A).

To expand this finding, a larger panel of HNSCC cell lines (CAL27, SCC040, FaDu, SCC47 and VU147) was exposed to a range of quinacrine concentrations. The resulting concentration-response curves illustrate that quinacrine effectively inhibits cell viability in a concentration dependent manner (Figure 1B) with IC_50_ values for cell lines tested ranging from 0.63 to 1.21 μM (Figure 1B and 1D), which is comfortably within clinically achievable concentrations [19–21]. These data indicated that quinacrine was a viable candidate for further development.

### Quinacrine increases the efficacy of cisplatin

**Figure 2:**
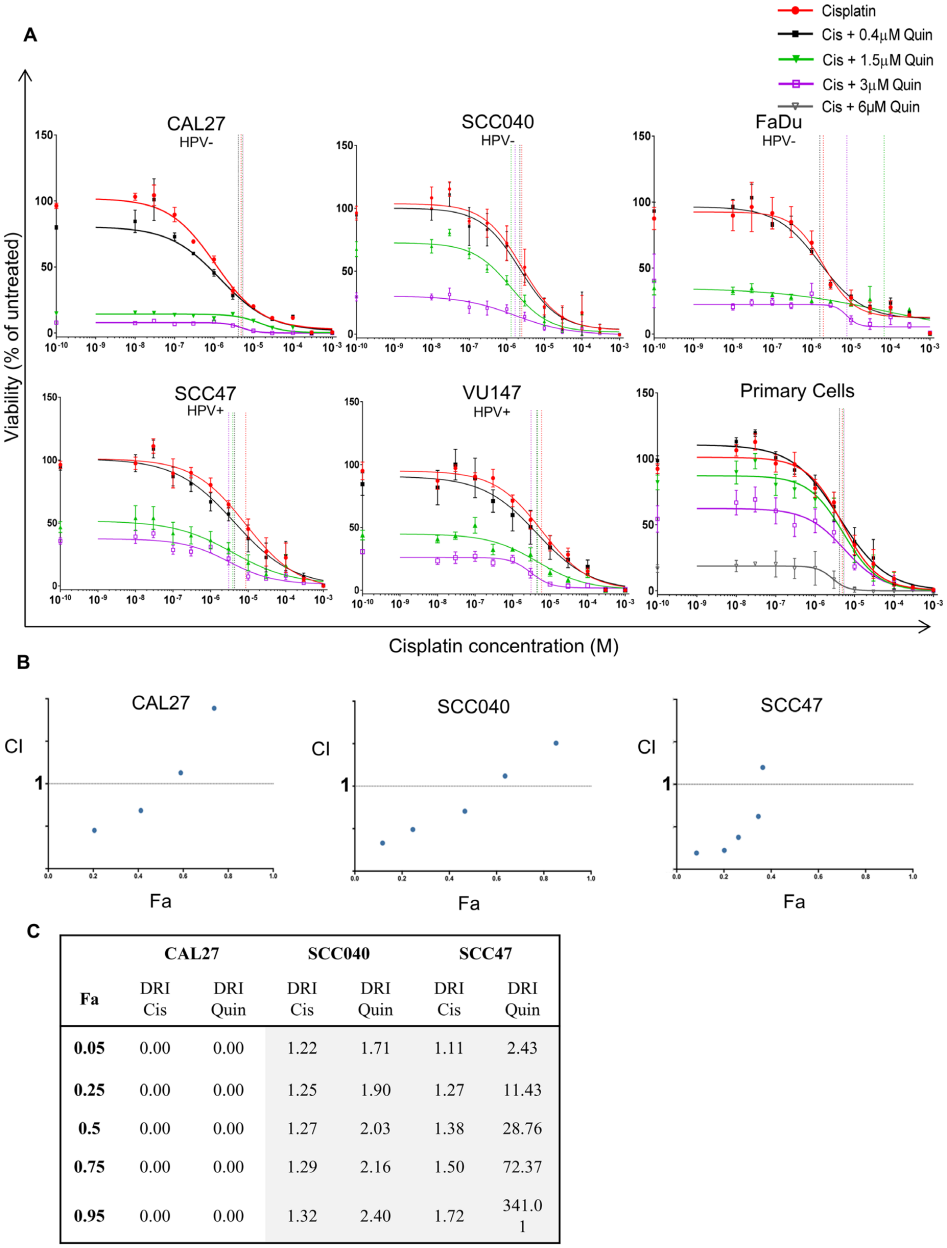
(**A**) Concentration response curves of cell lines (*n* = 3–4) and patient-derived primary tumor cells (*n* = 6) to increasing concentrations of cisplatin (red line) with the addition of 0.4 (black line), 1.5 (green line), 3 (purple line) or 6 μM (grey line) quinacrine. Vertical lines highlight IC_50_ values color matched; responses fitted to a five-parameter logistic equation. (**B**) Fraction affected vs Combination Index (Fa-CI) plot for each cell line, produced using CompuSyn. Concentrations range from 1/32x – 2x IC_50_ for quinacrine (Q), and from 1/8x – 8x IC_50_ for cisplatin (**C**), maintaining a ratio of 1:4 Q:C. Data points below 1 (dotted line) represent synergy (*n* = 3). (C) Dose reduction index (DRI) table for CAL27, SCC040 and SCC47 cell lines (*n* = 3). Green indicates DRI values > 1 (favorable reduction); orange indicates DRI values <1 (less favorable reduction).

HNSCC cell lines showed additional suppression of cell viability when quinacrine was combined with cisplatin (cell line IC_50_: 2, 3 or 10 μM), compared to quinacrine alone (Figure 1B). The combination of quinacrine and standard of care cisplatin was investigated further (Figure 2A). To demonstrate a concentration-dependent reduction in viability, our HNSCC cell lines were exposed to increasing concentrations of cisplatin, with and without the addition of quinacrine at 0.4, 1.5 and 3 μM. Quinacrine enhanced the ability of cisplatin to suppress cell viability in all cell lines. This reduction was more evident at lower concentrations of cisplatin, since cisplatin concentrations of 0.1 mM (10^–4^ M) or above resulted in dramatic suppression of viability of all cell lines, such that additional suppression by the addition of quinacrine was not possible. For example, when treated with a cisplatin concentration of 0.3 μM (3 × 10^–7^ M) alone, SCC040 showed cell viability suppression of 12%, compared to suppression of 17%, 48% and 79% following the addition of 0.4, 1.5 and 3 μM quinacrine to 0.3 μM cisplatin, respectively. In comparison, at a cisplatin concentration of 0.1 mM (10^–4^M), cell viability was reduced by cisplatin alone by 90%, with only marginal additional suppression by increasing doses of quinacrine.

### Quinacrine displays synergy with cisplatin

To confirm the above findings and to assess potential synergy of quinacrine when combined with cisplatin, Chou-Talalay analysis was undertaken [22]. Synergy was observed at lower concentrations of quinacrine and cisplatin, as demonstrated by a combination index (CI) number less than 1 (Figure 2B) when using a fixed ratio of quinacrine to cisplatin concentrations of 1:4 based on their IC_50_ values (IC_50_ values for quinacrine given in Figure 1D; cisplatin IC_50_ ~ 2 μM for CAL27 and SCC040, and 10 μM for SCC47). Dose reduction indexes (DRI) refer to the amount that one drug can be reduced by to maintain the same cell viability reduction, termed the fraction affected (Fa) (Figure 2C). DRI values higher than 1 indicate that one drug can be reduced by adding a second whilst achieving the same reduction in cell viability. For example, to achieve a 50% reduction in viability (Fa = 0.5) in SCC040 cells, the concentration of cisplatin can be reduced by 1.27 times when used in combination with quinacrine. Synergy appears to be reduced at higher cisplatin concentrations, likely due to the dramatic reduction in cell viability caused by high concentration cisplatin treatment alone.

### Quinacrine reduces cell viability of primary tumor cultures and potentiates the effects of cisplatin

Whilst cell lines are a good model, demonstration of anti-tumor activity against primary tumor cells direct from patients is much more valuable. Therefore, cells established from six HNSCC patients were cultured and treated at a range of quinacrine concentrations. All six primary patient HNSCC samples tested *in vitro* exhibited similar responses to quinacrine to those observed for the cell lines, with a mean IC_50_ of ~2 μM (Figure 1B and 1D).

When comparing the sensitivity of primary HNSCC cultures to the different cell lines at 1 μM quinacrine (10^–6^ M) (Figure 1C), primary cells exhibited cell viability suppression of 21.1%, similar to the SCC040 (14.3%) and VU147 (35.8%) cells lines, and lower than the CAL27 (72.3%), FaDu (56.7%) and SCC47 (47.3%) cells.

The primary cell cultures showed additional suppression of cell viability when 2 μM cisplatin was added to quinacrine (Figure 1B). As described in the cell lines above, increasing doses of quinacrine (0.4, 1.5, 3 and 6 μM quinacrine) enhanced the ability of cisplatin to suppress cell viability, more prominently at the lower doses of cisplatin (Figure 2A).

### Clonal survival is reduced by quinacrine

**Figure 3:**
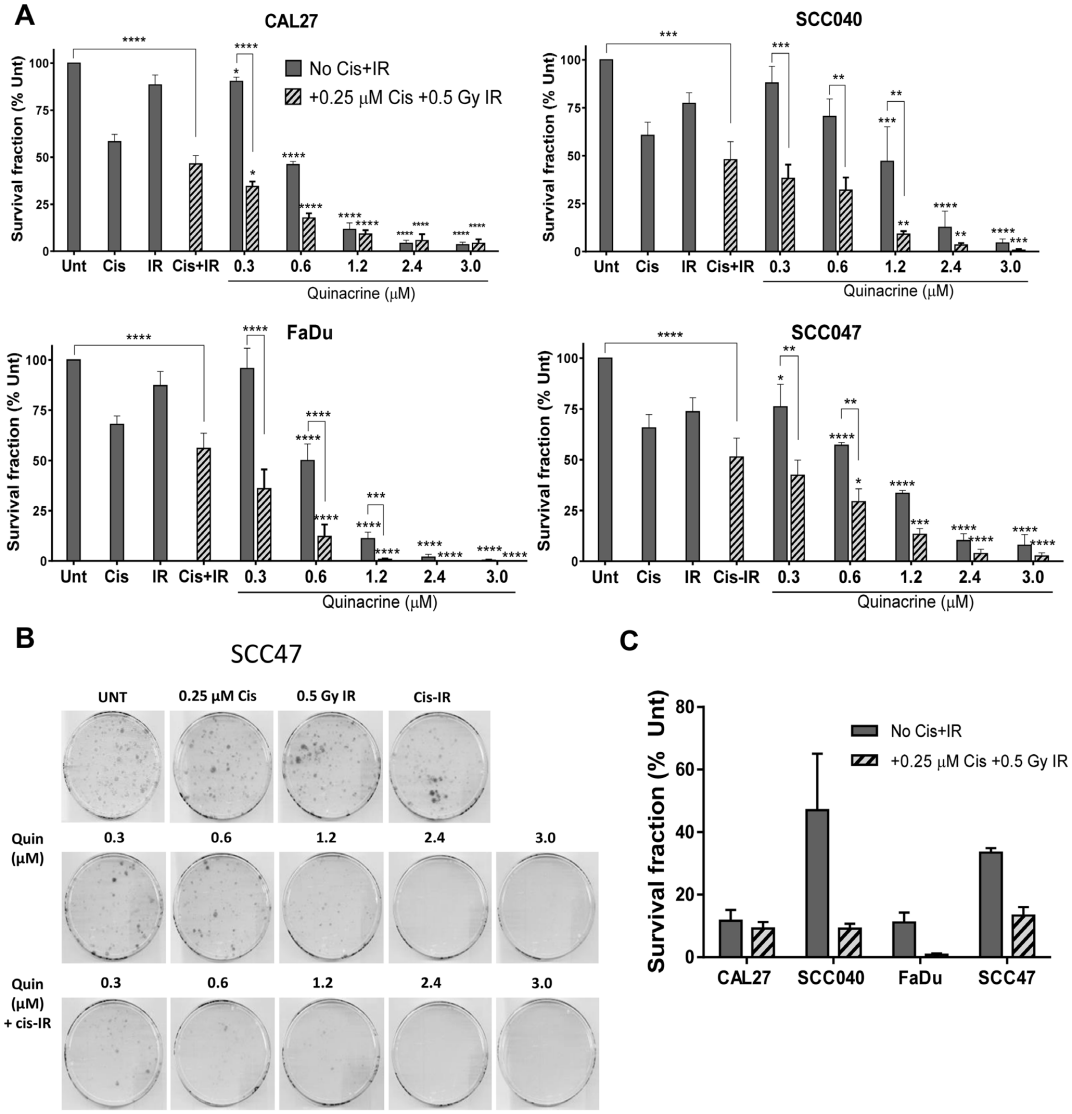
Clonogenic survival of cell lines exposed to increasing concentrations of quinacrine, with and without 0.25 μM cisplatin and 0.5 Gy irradiation (Cis+IR) for 24 hours (*n* = 3–4). (**A**) Values are presented as a percentage of untreated controls. A two-way ANOVA with Dunnett’s multiple comparison test was performed and *P*-values indicated on the graphs as follows: ^*^ *p* < 0.05, ^**^ *p* < 0.01, ^***^ *p* < 0.005, ^****^ *p* < 0.001. Stars directly above bars correspond with differences from their relevant control plates (Unt or Cis-IR). Stars above lines show differences between the treatments indicated. (**B**) Representative scanned plates showing SCC47 colonies surviving following treatment. (**C**) Relative survival of cells exposed to 1.2 μM quinacrine compared to their relevant control plates (Unt or Cis-IR), with and without Cis-IR.

Clonogenic assays were used to assess whether differences in viability were due to reduced clonogenic survival of HNSCC cells. All four cell lines (CAL27, SCC040, FaDu and SCC47) tested demonstrated a concentration-dependent reduction in survival fractions when treated with increasing concentrations (0.3, 0.6, 1.2, 2.4 and 3 μM) of quinacrine (Figure 3A and 3B). The relative sensitivities of the different cell lines to 1.2 μM quinacrine alone or in combination with cisplatin (0.25 μM) and irradiation (0.5 Gy) are displayed in Figure 3C. CAL27 and FaDu cells had the largest decrease in survival when treated with 1.2 μM quinacrine alone, reduced by 88.4% and 88.9% compared with untreated controls, respectively. SCC040 cells demonstrated 52.9% and SCC47 cells 66.6% reduced survival compared with controls.

Further reductions were also evident upon the addition of 0.25 μM cisplatin and 0.5 Gy irradiation (Cis-IR) to 1.2 μM quinacrine (Figure 3C). However, reductions in cell viability due to adding Cis-IR to quinacrine (adjacent bars on Figure 3A) only reached statistical significance (*p* < 0.05) at quinacrine concentrations equal to or lower than 1.2 μM. This is due to the large reductions in clonogenic survival caused by higher doses of quinacrine, such that radiotherapy and cisplatin could not cause significant additional reductions in survival.

### Quinacrine induces apoptosis

**Figure 4:**
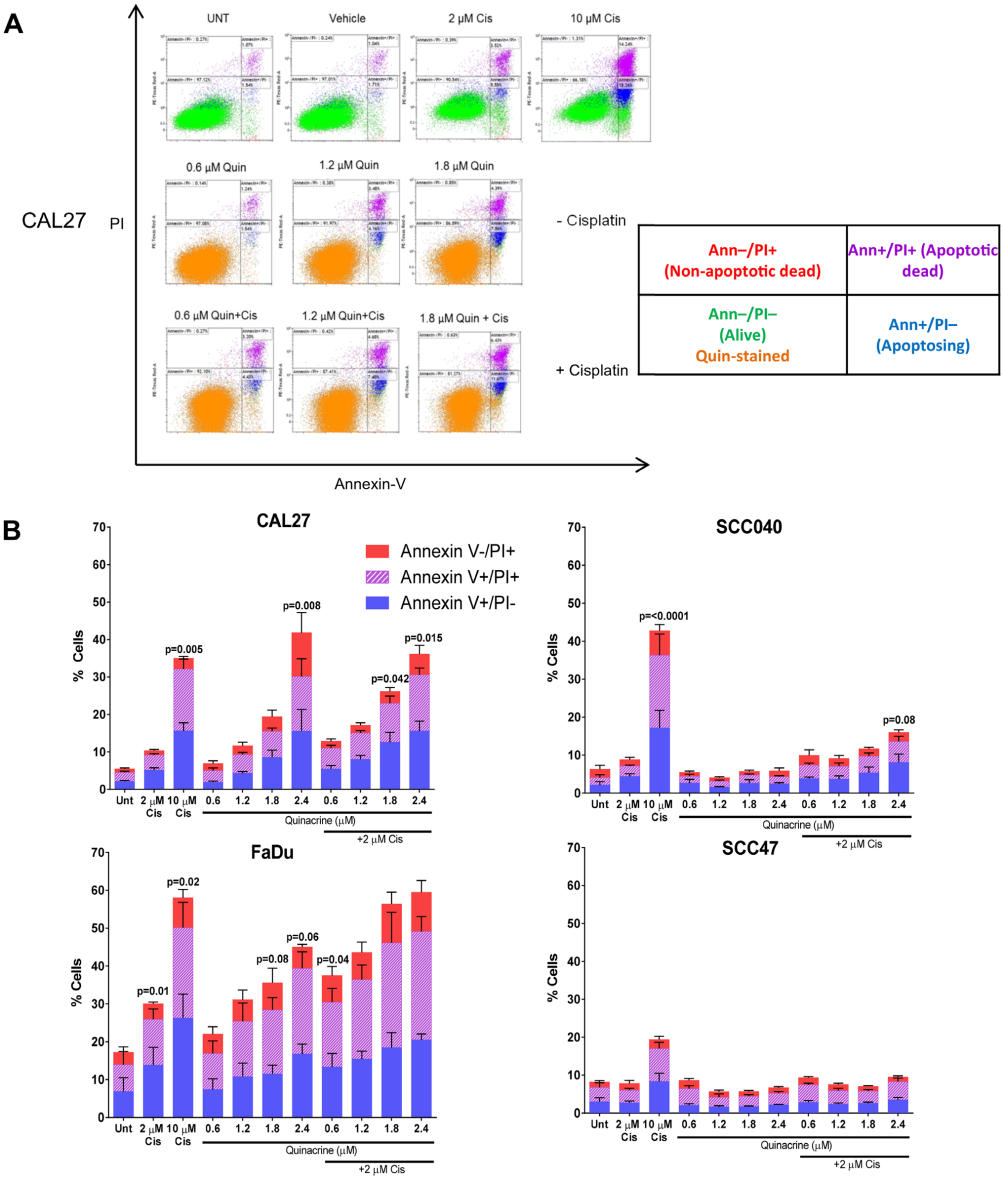
Assessment of cell death following 48 hour exposure to quinacrine +/- cisplatin. (**A**) Representative scatter plots highlighting different cell populations following treatment of CAL27 cells. The table describes cells within each quadrant. (**B**) Total apoptotic and dead cell populations were quantified using Kaluza software and split into Annexin V –ve/ PI +ve (non-apoptosing dead cells), Annexin V +ve/ PI +ve (dead through apoptosis) and Annexin V/ PI –ve (undergoing apoptosis). One Way ANOVA followed by Dunnett’s multiple comparisons test was performed between each treatment and the appropriate control (Untreated [UNT] or 2 μM Cisplatin [Cis]) (*n* = 3–6).

To understand the mechanisms of quinacrine-induced cell death in HNSCC cell lines, we first investigated apoptosis using the Annexin V apoptosis assay. The table in Figure 4A demonstrates how various groups of live, dead and dying cells were identified by flow cytometry. Cells that are alive (within the lower left quadrant) became slightly more dispersed following the addition of quinacrine (32) compared to those not receiving quinacrine (green). This is caused by the DNA-intercollation and subsequent auto-fluorescence of quinacrine within the FITC channel. These qualities do not interfere with assessment of apoptotic and non-apoptotic cell death, as quinacrine emits light at 488 nm, which is spectrally distinct from PI fluorescence (610 nm) and Annexin V-Cy5 fluorescence (680 nm).

CAL27, SCC040, SCC47 and FaDu cells were exposed to cisplatin alone (2 μM and 10 μM), increasing concentrations of quinacrine (0.6, 1.2, 1.8 and 2.4 μM) alone or a combination of increasing quinacrine concentrations and 2 μM cisplatin for 48 hours prior to Annexin V/PI quantification. CAL27, FaDu and, to a lesser extent, SCC040 cells showed a dose-dependent increase in Annexin V positive and PI positive cells with increasing quinacrine concentrations (Figure 4B). FaDu cells showed the largest increase in apoptosing cells and apoptotic cell death when exposed to quinacrine, with 39.4% of cells displaying Annexin V positivity when treated with 2.4 μM quinacrine alone and 49.1% positive when treated with 2.4 μM quinacrine in combination with 2 μM cisplatin. CAL27 cells also showed considerable apoptosis following 2.4 μM quinacrine treatment alone, with 30.1% cells positive for Annexin-V. Interestingly, SCC47 cells did not show sensitivity towards quinacrine or 2 μM cisplatin alone, reflecting the diversity in cell lines utilized.

### Autophagic flux is altered by quinacrine treatment

**Figure 5:**
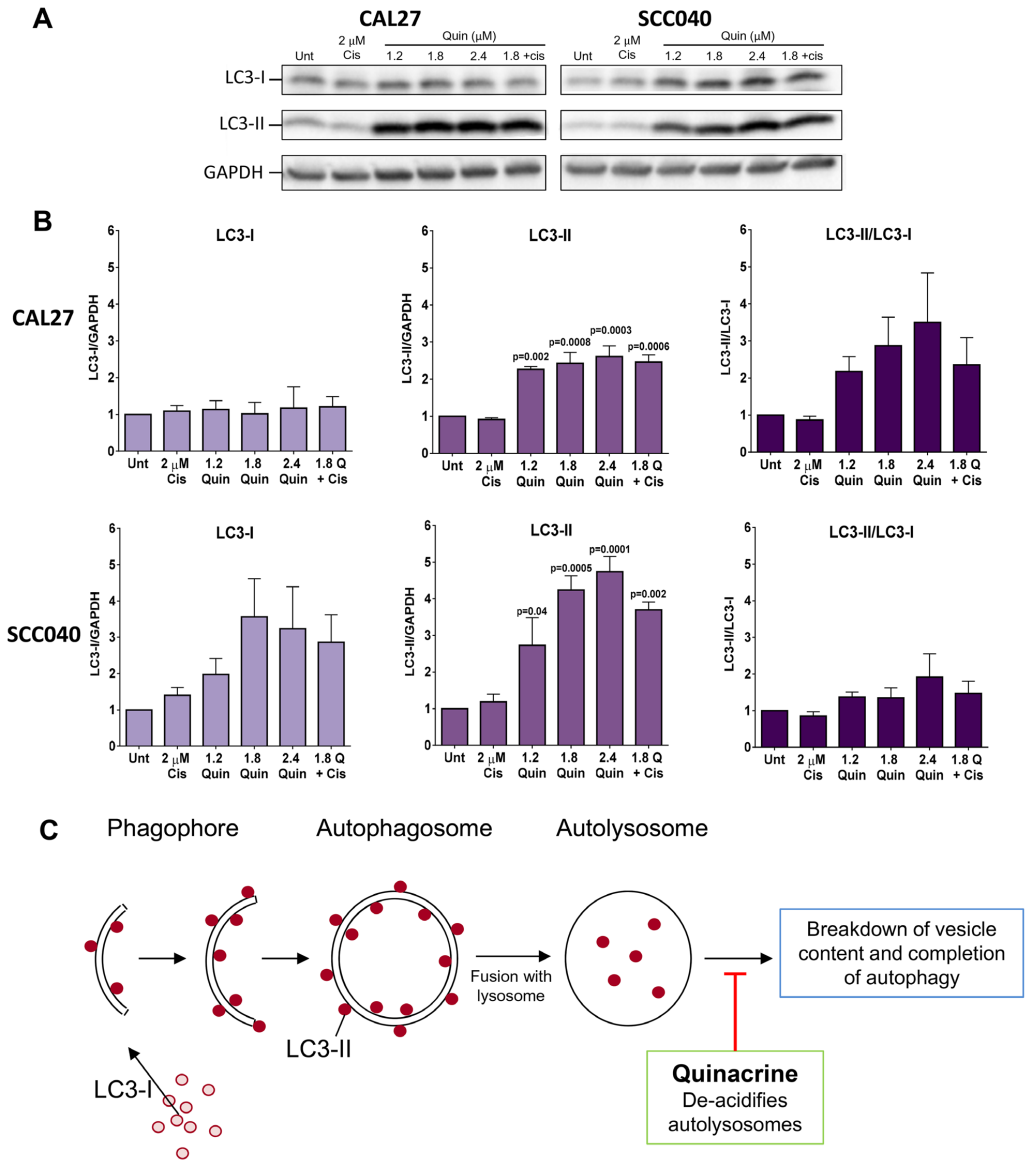
Western blot analysis of autophagy induction. (**A**) Representative Western blots showing levels of LC3-I and LC3-II in CAL27 and SCC040 cells following 48 hours exposure to quinacrine at increasing concentrations (1.2, 1.8 and 2.4 μM), 1.8 μM quinacrine with 2 μM cisplatin and 2 μM cisplatin alone. GAPDH was used as a loading control. (**B**) Histograms from ImageJ densitometry analysis representing the quantitative changes in LC3 levels in each of the cell lines. LC3-II/LC3-I demonstrates the relative levels of the two proteins. Each band was normalized to an internal untreated control band. *P*-values above bars highlight differences vs untreated control cells following one-way ANOVA followed by Dunnet’s multiple comparisons test (*n* = 3). (**C**) Schematic showing the recruitment of LC3-II to autophagosomal membranes and the ability of quinacrine to exert its inhibitory affects and prevent completion of autophagy.

The ability of quinacrine to mediate autophagy has been shown in other cancer types, but not in HNSCC to date. We therefore analyzed LC3 levels to determine whether quinacrine affects autophagic flux within CAL27 and SCC040 cells (Figure 5). LC3-I is constitutively expressed within the cytosol and converted to LC3-II upon recruitment to autophagosome membranes; therefore LC3-I/LC3-II is often utilized as a marker of autophagy initiation. LC3-II is later degraded within autolysosomes upon completion of autophagy [23]. Quinacrine has been shown to sequester hydrogen ions within autolysosomes, which raises pH and impairs the breakdown of cellular components and therefore inhibits completion of autophagy, leading to a build-up of LC3-II [15] (Figure 5C).

Cells were exposed to cisplatin alone, quinacrine at 1.2, 1.8 and 2.4 μM alone or 1.8 μM quinacrine plus 2 μM cisplatin for 48 hours. Expression of LC3-I and LC3-II was then determined by Western blotting. Both cell lines displayed a dramatic increase in LC3-II at all concentrations of quinacrine treatment tested (Figure 5A–5B), whereas LC3-I remained unchanged in CAL27 cells and showed a non-significant increase in SCC040 cells. The accumulation of LC3-II in this setting is therefore indicative of quinacrine inhibiting the completion of autophagy. In contrast, cisplatin addition did not significantly alter levels of LC3-I or LC3-II in either cell line tested.

We assessed p53 expression in both SCC040 and CAL27 cell lines following 48 hours exposure to quinacrine (1.2, 1.8 and 2.4 μM) alone and with 1.8 μM quinacrine + 2 μM cisplatin to assess the potential influence of p53 on apoptosis in these cell lines. CAL27 cells possess a mutation in p53, rendering it non-functional [24]. They demonstrated no change in expression of p53 or the downstream protein p21 following quinacrine or cisplatin treatment (Supplementary Figure 1). SCC040 cells express wild-type p53 [25]. Again, no change in p53 or p21 expression was seen as a result of quinacrine or cisplatin treatment after 48 hours (Supplementary Figure 1) This would indicate that the mechanisms of action of quinacrine, within our experimental setup, are likely to be independent of TP53 status and p53 function.

### Tumor xenograft growth is impaired by quinacrine and cisplatin

**Figure 6:**
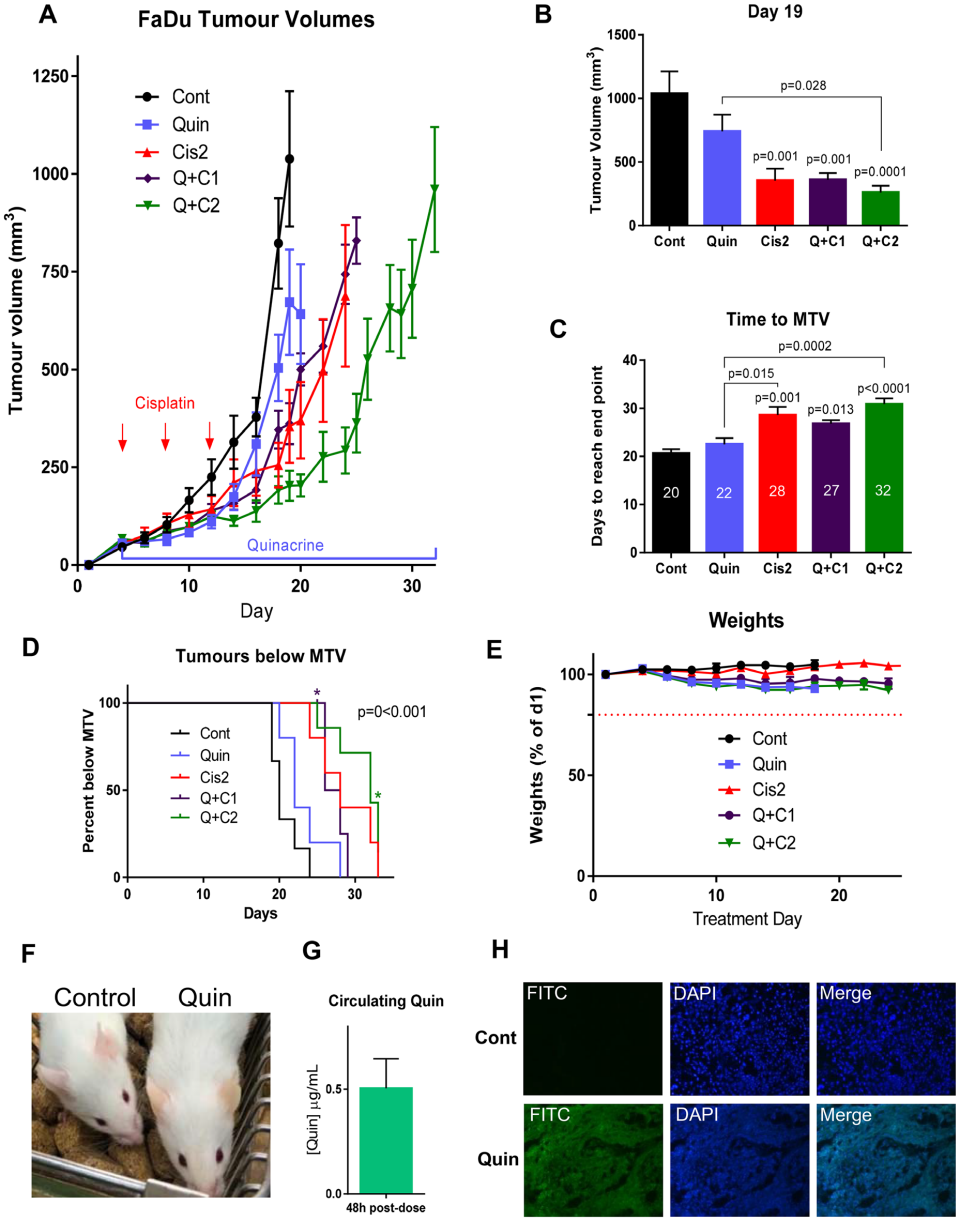
(**A**) Tumor growth in mice bearing FaDu tumors treated with 100 mg/kg quinacrine and or cisplatin (1 or 2 mg/kg, as indicated), *n* = 5–6 per group. (**B**) Tumor volumes on day 19 (*n* = 5–7 per group). (**C**) Time taken for tumors to reach maximum tumor volume (MTV) with median days for each treatment indicated on bars. (**D**) Kaplan-Meier plot showing the proportion of animals with tumors below MTV over time. ^*^Star indicates a quinacrine + 2 mg/kg cisplatin animal being culled for reasons other than tumor size (*n* = 5–7 in each group). A log-rank (Mantel-Cox) test showed a significant difference between groups, represented on the graph. (**E**) Weights of mice over the experiment. (**F**) Slight yellowing of the skin caused by quinacrine treatment (right) next to a vehicle-treated animal (left). (**G**) Concentrations of quinacrine in mouse plasma 48 hours post-gavage treatment on day 19 of treatment (*n* = 5). (**H**) Cross sections of representative tumor sections taken at the end of the experiment from vehicle and quinacrine-treated mice showing autofluorescence in the FITC channel of cells incorporating quinacrine into their DNA (*n* = 3).

To substantiate the findings in the HNSCC cell lines and in patient-derived tumor cells, NSG mice bearing FaDu xenograft tumors were treated orally with 100 mg/kg quinacrine every two days, with and without 1 mg/kg or cisplatin (2 mg/kg or 1 mg/kg) via intraperitoneal injection on days 4, 8 and 12. Tumor volumes at day 19, when the first control animal was culled, show reductions in the size of treated tumors, mainly in those treated with quinacrine and 2 mg/kg cisplatin (*p* = 0.0001) (Figure 6A and 6B).

Quinacrine alone showed early inhibition of tumor growth, and modestly extended the mean time needed to reach maximum volume by 2 days (from 20 to 22 days), compared to untreated control (Figure 6A and 6C). In comparison, higher dose cisplatin (2 mg/kg) alone extended the mean time to reach maximum tumor volume by 8 days, from 20 to 28 days compared to untreated control. Quinacrine was particularly effective when combined with high dose cisplatin, whereby the time taken for tumors to reach their maximum volume could be extended by 12 days, taking a median of 32 days compared to 20 days for untreated controls to reach a tumor volume of 1000 mm^3^ (*p* < 0.0001), and was also significantly higher than the time taken to reach maximum tumor volume in the group treated by quinacrine alone, from 22 days to 32 days (*p* = 0.0002). There was a difference in the time needed to reach maximum tumor volume between the combined quinacrine and 2 mg/kg cisplatin group and the 2 mg/kg cisplatin alone group of 4 days, however this did not reach statistical significance (*p* = 0.6), possibly due to the small sample size and corrections for multiple comparisons.

The Kaplan-Meier plot (Figure 6D) further emphasizes the extended time for treated tumors to reach their maximum tumor volume (MTV) when mice were treated with cisplatin (2 mg/kg) and quinacrine, even though one animal was culled early due to a deterioration of their overall condition and not due to tumor volume. The Hazards ratio for time taken to reach MTV in the 2 mg/kg cisplatin and quinacrine group compared to the 2 mg/kg cisplatin alone group was 0.4 (95% CI, Mantel-Haenszel test).

Importantly, the *in vitro* experiments demonstrated that the synergy between quinacrine and cisplatin was highest at lower doses of cisplatin, where quinacrine potentiated the effect of cisplatin most. At higher concentrations of cisplatin tumor kill was so high that there was little possibility for quinacrine to add an effect. We therefore also tested the combination of quinacrine with a lower dose of cisplatin (1 mg/kg – half the higher dose used). At that concentration, quinacrine and 1 mg/kg cisplatin resulted in the same growth rate as 2 mg/kg cisplatin alone, indicating that by adding quinacrine, the dose of cisplatin can be halved whilst maintaining the same anti-cancer efficacy, which could have significant benefits to patients by reducing toxicity related side effects.

Quinacrine and cisplatin were well tolerated throughout the *in vivo* experiment. The weights of mice remained well within the 20% weight loss cut-off (Figure 6E). A slight yellowing of the skin of animals treated with quinacrine was apparent due to the auto fluorescent properties of the drug (Figure 6F).

### Quinacrine is present in plasma and tumor tissue

Quinacrine was administered at 100 mg/kg to mice every 48 hours via oral gavage. To establish whether a steady, therapeutic concentration was maintained within the circulation of mice receiving oral quinacrine treatment, blood samples were taken 48 hours after the 9th dose of quinacrine (on day 19 of the toxicity experiment). Analysis carried out by LC-MS (Supplementary Figure 2) revealed that even 48 hours after the last dose, plasma concentrations of 0.5 mg/mL were still present (Figure 6G), equating to 1.25 μM, a dose that had resulted in a significant response using the alamarBlue^®^ viability assay in all cell lines tested (Figure 1B–1D) and initiated apoptosis in CAL27 and FaDu cells (Figure 4B).

To establish whether quinacrine successfully entered tumors from the blood, sections were taken and quinacrine fluorescence visualized at 488nm. Tumor samples from mice receiving quinacrine treatment showed clear fluorescence throughout their tumors, whereas those receiving only control treatment did not (Figure 6H); thus confirming that quinacrine is able to reach tumor tissue following oral administration.

## DISCUSSION

Our data support the use of quinacrine as a potential treatment option for HNSCC. We have shown that *in vitro* quinacrine (at clinically relevant concentrations) reduces viability of head and neck cancer (HNC) cell lines and primary cultures, decreases clonogenic survival, increases apoptosis and inhibit autophagy. Importantly, effects were in addition to those resulting from standard treatment (cisplatin +/– irradiation), and importantly we have demonstrated synergy of quinacrine with cisplatin.

Quinacrine’s ability to promote apoptosis of cancer cells has been previously reported and mechanisms for this have been suggested. Firstly, it is thought that quinacrine enhances the binding of the TRAIL ligand to Death Receptor 5 (DR5) via formation of a stable quinacrine-TRAIL-DR5 complex [26]. In addition to this, quinacrine has been shown to restore deficient, wild-type p53 function in UM-SCC HNSCC cell lines by inducing TP53 mRNA and protein expression. Hence, as p53 can regulate cisplatin-induced cell death by several mechanisms, this increased their sensitivity to cisplatin [14]. The ability of quinacrine to restore p53 function would be promising in HNC as the majority of HNSCC that retain wild-type TP53 genotype have previously been shown to exhibit low immunostaining for TP53 protein [27]. SCC040 cells express wild type p53, whilst CAL27 cells express mutant p53 (p.H193L). In our study, no change in p53 expression or its downstream protein p21 was observed in CAL27 and SCC040 cells following 48 hours of quinacrine exposure. This may be because both cell lines expressed sufficient levels of p53 at baseline. Mechanisms independent of p53 are therefore likely to be driving the majority of apoptosis within these two cell lines.

The divergent proportions of apoptosis seen between the HNC cell lines in this study may be due to intrinsic differences in the expression of proteins within the apoptotic pathway. For example, the poor prognostic marker and anti-apoptotic protein, MCL1 (Myeloid Cell Leukemia Sequence 1 [BCL2-related]), is a target of quinacrine, as shown in anaplastic thyroid cancer [28]. Low MCL1 expression, as in non-malignant tissue, corresponds with lack of response to drug treatment.

Quinacrine has previously been shown to cause the build-up of dysfunctional autolysosomes as evidenced by accumulated LC3-II [29]. Our study supported these findings with dramatic increases in LC3-II protein expression in HNSCC cell lines following quinacrine exposure. In ovarian cancer, disruption of autophagy sensitizes cells to autophagic cell death and apoptosis via a p53-independent pathway when exposed to cisplatin [17]. We hypothesize that quinacrine’s ability to impede autophagy increases stress within HNSCC cells, thus inducing apoptosis.

*In vivo* quinacrine treatment was well tolerated and data presented here is the first to show that quinacrine, at clinically achievable concentrations is able to reduce tumor burden in mice, extending median time to reach maximum tumor volume. Analysis of plasma concentrations levels of quinacrine during these studies showed a steady state blood plasma concentration of around 0.5 mg/ml (~ 1.25 μM). This is a concentration at which all cells tested *in vitro* were responsive to quinacrine, including primary tumor cells from HNSCC patients. Due to its relatively long half-life (5–14 days) quinacrine is maintained at a therapeutic dose 48 hours after dosing, indicating a large treatment window allowing for flexible dosing schedules [30].

Importantly, our *in vivo* data has shown that quinacrine is able to penetrate the target tissue following oral administration. Drug, also, accumulated in other tissues, including the skin and liver, causing some yellow discoloration; this has been reported previously and is cleared rapidly following cessation of quinacrine treatment [11]. We demonstrated that combination of quinacrine with cisplatin improved tumor growth inhibition compared to quinacrine or cisplatin alone. Importantly, the combination of quinacrine with cisplatin enabled the dose of cisplatin to be halved, whilst maintaining the same impairment of tumor growth.

Extensive, long-term data on safety of quinacrine is available due to of its widespread use by United States soldiers during the Second World War; three million people treated with the drug for up to four years [30]. These data show that quinacrine is safe and lacks serious side-effects. Low toxicity in non-cancerous tissue is extremely beneficial when identifying novel cancer treatments, as adverse off-target effects often limit the use of toxic chemotherapeutics; quinacrine has previously been demonstrated to have very low toxicity in normal mononuclear cells [31]. Quinacrine has been tolerated well in humans at doses up to 800 mg/day for a 70 kg adult [11]. The dose used in our *in vivo* efficacy study of 100 mg/kg equates to a human dose of 568 mg, which is well within the levels clinically tolerated [11, 32]. Four early phase clinical trials have been initiated since 2006 examining the safety and efficacy of quinacrine in the treatment of lung (NCT01839955), colorectal (NCT01844076), renal (NCT00574483) and prostate cancer (NCT00417274), of which three are complete and one has reported results [33]. In the latter study, all patients completed treatment, indicating the tolerability of quinacrine.

Cisplatin is a highly toxic chemotherapeutic agent, which can cause severe nausea and vomiting, renal failure and cardiovascular damage, among other side-effects [34]. Therefore, reducing and replacing cisplatin use, even partially, whilst maintaining efficacy could be of considerable benefit to patients. This is especially relevant in older or unfit patients, who currently may be excluded from receiving cisplatin at current doses due to its poor tolerability. It is also particularly important as up until recently cetuximab was used as an alternative to cisplatin in less fit patients with HNSCC. However, two recent randomized studies have demonstrated that cetuximab is not only as toxic as cisplatin, but is also less effective in the treatment of HPV+ HNSCC [3, 4], and therefore there is an ongoing unmet need for treatments with lower toxicity.

Our study has an intrinsic limitation that all *in vitro,* and *ex vivo,* studies have as the cells are studied in artificial environments, resulting in the disruption of some interactions that regulate biological activity. In order to address these limitations, we also tested the efficacy of quinacrine *in vivo,* using a HNC xenograft mouse model. We used subcutaneous xenograft mouse models due their reproducibility, reliable assessment of tumor growth and response to treatment. However, all mouse models have well recognized limitations as human cancer models [35].

The data presented in this paper, combined with evidence from additional cancer types, support the study of quinacrine in addition to standard chemotherapy for improving outcome in HNSCC patients, especially with the aim of using the combination in patients who cannot tolerate the full dose of cisplatin. Quinacrine with its wealth of safety data reduces the potential risk to patients and clinical trial sponsors, therefore making it an appealing candidate to move forward into clinical assessment with cisplatin. We are in the process of initiating a clinical trial in HNSCC patients for the assessment of quinacrine as an anti-cancer treatment.

### Significance statement

Head and neck cancer (HNC) patients have limited treatment options, often associated with severe, potentially life-threatening, side effects. We demonstrated efficacy of quinacrine and synergy with standard-of-care cisplatin against HNSCC *in vitro*. Notably, our study is the first to demonstrate that quinacrine is able to reduce tumor burden in mice bearing HNSCC xenograft tumors, using concentrations that are clinically relevant. Importantly, combination with quinacrine enabled the dose of cisplatin to be halved in mice, whilst maintaining the same impairment of xenograft tumor growth. As quinacrine has little toxicity, this suggests potential therapeutic benefit in older and frail patients.

## MATERIALS AND METHODS

### AcceleraTED drug repurposing platform

At the Institute of Head and Neck Studies and Education (InHANSE), we have developed the ‘AcceleraTED’ drug repurposing platform. It aims to assess the efficacy of libraries of Food and Drug Administration/ European Medicines Agency (FDA/EMA)-approved drugs as anti-cancer agents against HNSCC, with the aim of rapidly progressing promising candidates into the clinic.

The platform consists of five main stages, each with go/no go criteria to allow progression of a hit to the next stage: The first stage utilizes a high-throughput alamarBlue^®^ (ThermoFisher Scienctific) cell viability screen to identify compounds that suppress viability of HNSCC cell lines. We use a cut off of 50% suppression of viability or more when the drug is used at clinically-achievable concentrations [C_max_ (maximum serum concentration observed in patients) or lower]. Stage two validates the findings of the initial screen using a panel of well-characterized HNSCC cell lines and then assesses concentration responses, clonogenic survival, and the ability of compounds to induce apoptotic cell death and autophagy. Stage three further validates results by assessing concentration-response curves using primary cell cultures grown directly from HNSCC patients. The fourth stage undertakes *in vivo* testing, whereby initial toxicity is established, followed by evaluation of efficacy against HNSCC xenograft tumors. Following successful completion of these stages, stage five intends to test the safety of compounds in combination with standard treatment in early-phase clinical trials.

More detail on individual assays is given below. All experiments represent a minimum of at least three independent repeats, as indicated.

### Drugs and chemicals

All drugs and chemicals were sourced from Sigma (now Merck) unless otherwise stated. Concentrations used were based upon clinically-achievable concentrations described previously in the literature based on clinically prescribed doses. Solubility information was taken from documentation accompanying the drug library or PubChem.

### Cell cultures

The human papillomavirus (HPV) negative cell lines CAL27 (ACC-446; DSMZ), SCC040 (UPCI-SCC-040; ACC-660; DSMZ) and FaDu (ATCC HTB-43), and HPV positive cell lines SCC47 (UM-SCC-47; Millipore) and VU147 (gifted from Prof. J. de Winter, Amsterdam, Netherlands) were used. The cell lines were cultured using Dulbecco’s Modified Eagle’s Medium-HEPES Modification (DMEM) supplemented with 10% fetal bovine serum (FBS), 1% L-glutamine, 1% penicillin/ streptomycin and 1% non-essential amino acids (NEAA) at 37°C. All cell lines were verified mycoplasma negative every six months, along with annual authentication via Short Tandem Repeat (STR) profiling.

### Patient-derived primary cell cultures

Tumor biopsies were obtained from HNSCC patients undergoing diagnostic biopsy or tumor resections, and who were consented under the Human Research Authority (HRA) Human Tissue Act 2004 ethics approval number 16/NW/0265. Tissue samples were collected and within 30 minutes washed five times in transport medium (DMEM with HEPES modification supplemented with 10% FBS, 2 mM L-glutamine, 50 μg/ml penicillin/ streptomycin, 50 μg/mL gentamicin, 10 μg/mL clindamycin, 5 μg/ml amphotericin B and 5 μg/mL chloramphenicol). Following washing, the tissue was disaggregated using sterile scalpels, transferred to serum-free media (SFM) (as part of a kit including BPE and EGF), supplemented with 50 μg/mL penicillin/ streptomycin, 50 μg/ml gentamicin, 5 μg/mL amphotericin B and 10 μM ROCK inhibitor (Y-27632) (Bio-techne, Tocris) and grown on rat tail collagen (8 μg/mL) coated plates at 37°C. After 24 hours, media was replaced and cells left for 7 days to assess growth.

### Cell viability and proliferation

The alamarBlue^®^ (Invitrogen) cell viability assay was utilized to measure the relative viability of cells as a measure of proliferation. AlamarBlue^®^ contains a blue dye called resazurin that changes to a pink, highly fluorescent form in the presence of metabolically active cells, giving a quantitative measure of the relative number of viable cells. Cells were seeded in 96 well plates at pre-optimized densities. After 24 hours, appropriate treatments were added to wells and cells were incubated for a further 68 hours (cell lines) or 92 hours (patient-derived primary cells). These time differences reflect the growth rate of cells and allow for the time required to undergo two cell divisions. Cells were then incubated for 4 hours with 20 μL (10% of final volume) alamarBlue^®^ reagent per well. Fluorescence was then measured at 550 nm. All relative fluorescence unit (RFU) values were normalized by deducting values from paired plates containing only media and drug (but no cells) and plotted as a percentage of untreated. All assays were carried out in triplicate and the mean values calculated.

### Initial drug screen

The Prestwick Chemical Library^®^ was used for the initial screen for this study. The library consists of 1280 FDA/EMA approved, off-patent drugs [36]. For the initial, high-throughput screen, drugs were tested at 10 μM concentrations using the alamarBlue^®^ cell viability assay on at least 2 HNSCC cell lines for each drug.

Those drugs achieving at least 50% suppression of cell viability in both cell lines were considered promising and subsequently analyzed in a secondary confirmatory screen using C_max_ (or lower) values in alamarBlue^®^ cell viability assays using CAL27 (HPV-) and VU147 (HPV+) HNSCC cell lines. Again, hits were confirmed as compounds that reduced cell viability by 50% or more compared to untreated controls.

### Concentration curves

Concentration-response curves were generated for quinacrine for each of five HNSCC cell lines and on patient-derived primary cells, following treatment with both quinacrine alone and in combination with cisplatin using alamarBlue^®^. In addition, response to increasing cisplatin concentrations in combination with 0, 0.4, 1.5, 3 and 6 μM quinacrine was also assessed. Each experiment was carried out in triplicate and normalized to untreated control wells containing only media and drug.

### Synergy experiments

CAL27, SCC040 and SCC47 cells were plated in 96 well plates and exposed to fixed ratios of quinacrine and cisplatin in relation to their IC_50_ values. Cells were exposed to drugs for 72 hours and cell viability was quantified using alamarBlue^®^. The optimal ratio was found to be a 1 to 4 ratio of quinacrine to cisplatin. Chou-Talalay analysis was then carried out using CompuSyn software (Nick Martin, MIT, Cambridge, MA). This calculates the fraction affected (Fa), a combination index (CI) and dose-reduction index (DRI). In our experiments, the dose reduction index refers to how many times the dose of one drug can be reduced when in combination with another drug and still elicit the same efficacy. A combination index value less than one is indicative of a synergistic effect; a value of one infers additivity and values above one suggest sub-additive responses.

### Cell survival and proliferation

To assess the survival of HNSCC cells and their ability to proliferate to produce clonal colonies following 24 hour drug exposure, clonogenic assays were utilized. CAL27, SCC040, FaDu and SCC47 cells were plated at optimized seeding densities in 10 cm plates and treated 24 hours later with a range of quinacrine concentrations +/– 0.25 μM cisplatin and 0.5 Gray irradiation. Culture medium was replaced after a further 24 hours and then the cells were left to establish colonies in the absence of any treatment. After 10–14 days depending on the cell line, colonies were stained using crystal violet (0.5% w/v), containing glutaraldehyde (6% v/v). Colonies were counted using Image J software. Due to the nature of growth of SCC47 growth, with less dense colonies, plates could not be analyzed on Image J and were counted manually. Plating efficiency was calculated by dividing the number of colonies formed by the number of cells seeded, which was then used to determine the survival fraction of each treatment normalized to the untreated control.

### Apoptotic cell death analysis using Annexin V/propidium iodide flow cytometry

The ability of quinacrine to induce apoptosis was investigated using Annexin V/ propidium iodide (PI) assays. The Annexin V antibody binds to phosphatidylserine on the outer surface of cells undergoing apoptosis. PI is added to highlight all dead cells, regardless of mechanism of cell death. CAL27, FaDu, SCC040 and SCC47 cells were seeded in 6 well plates at the following number of cells per well: 1^5^ × 10^5^ CAL27; 1 × 10^5^ SCC040; 1.4 × 10^5^ FaDu; 3 × 10^5^ SCC47. Drug treatments were added 24 hours later. After 48 hours incubation, cells were harvested and analyzed using Annexin V-Cy5 (PromoCell, Heidelberg, Germany) and the Dead Cell Apoptosis Kit (ThermoFisher Scientific, MA, USA) as per manufacturer’s instructions. The percentage of apoptotic cells was determined by Annexin V-Cy5 positivity using flow cytometry. From each sample, 30,000–50,000 events were recorded using an LSR II flow cytometer (BD Biosciences, CA, USA). Quantification as a percentage of the overall live and dead cell populations was carried out using Kaluza Software (Beckman Coulter, CA, USA).

### Western blot immunoreactivity analysis

To investigate the effect that quinacrine exerts on autophagy, protein expression of the autophagy marker, Microtubule-associated proteins 1A/1B Light Chain 3B (LC3), was assessed by immunoreactivity Western blotting. All reagents were obtained from BioRad unless otherwise stated. Expression of the tumor suppressor protein, p53, was also visualized. Ten cm plates were seeded with 1.2 × 10^6^ CAL27 or SCC040 cells per plate. After 24 hours, the appropriate drugs were added to the plates. Forty-eight hours after the addition of drugs, cells were harvested in RIPA buffer (Thermo Scientific) supplemented with protease and phosphatase inhibitors and EDTA (Thermo Scientific). Membranes were exposed to primary antibodies diluted in 5% BSA-TBS-T as follows: LC3 1:500 (Novus Bio, NB100-2331); β-actin 1:5000 (Abcam ab15580); p53 1:1000 (Santa Cruz, sc-126); p211:1000 (Santa Cruz, sc-53870). Following overnight incubation, membranes were exposed to anti-rabbit secondary antibody 1:5000 (Promega, W4011) or anti-mouse secondary antibody 1:1000 (DAKO, P0447) as appropriate and imaged using a ChemiDoc Imaging system. Densitometry was carried out using ImageJ software. LC3-II accumulation following treatment was used to indicate the impairment of cells to complete autophagy and therefore accumulate autophagosomal markers.

### *In vivo* efficacy study

In the fourth, *in vivo* validation stage of the AcceleraTED platform, we examined overt toxicity and the anti-tumor efficacy of quinacrine, alone and in combination with cisplatin. Male NOD/SCID/gamma (NSG) mice (Charles River) were kept in 12 hour light and 12 hour dark cycles in individually ventilated cages. All procedures were carried out in accordance with the UK Home Office Animal (Scientific Procedures) Act 1986 and approved by the local University of Birmingham Ethical Review Committee. Mice were maintained on mouse feed and water, ad libitum, and were at least 6 weeks old at the start of treatment.

NSG male mice were implanted with 5 × 10^6^ FaDu HNSCC cells, suspended in serum-free medium, by subcutaneous injection into the right flank. Tumors were given three days to become established, at which point mice were randomly allocated into five treatment groups as follows: (1) control (PBS); (2) 200 mg/kg quinacrine dissolved in PBS via daily oral gavage from day 4 onwards; (3) 2 mg/kg cisplatin dissolved in PBS given via intraperitoneal (IP) injection on days 4, 8 and 12; (4) 1 mg/kg cisplatin with 200 mg/kg quinacrine as in groups two and three; (5) 2 mg/kg cisplatin with 200 mg/kg quinacrine as in groups two and three. Animals were monitored daily for signs of ill health. Tumor measurements were taken using calipers and volumes calculated using the formula L × W^2^.

Mice were culled once tumors reached a maximum of 1250 mm^3^. Tumors were then excised and paraffin-embedded or frozen for subsequent analysis. Each group contained 6–8 animals. When displaying tumor growth over time, to gain an understanding of efficacy over a longer period, data was plotted until at least 5 animals per group remained. When fewer animals remained in a group, no more data were plotted. Data from all animals, however, were included when summarizing tumor volumes at day 19 and displaying time taken to reach maximum tumor volume (MTV).

### Circulating quinacrine quantification

To quantify minimum steady state plasma levels of quinacrine, up to 100 μL blood was taken from the tail vein of each animal 48 hours after the last oral dose at the end of the second week of dosing during toxicity experiments. Samples were spun for 10 minutes at 123 × g to separate the plasma, which was stored at –80ºC until analysis.

### Blood analysis for circulating quinacrine using HPLC-MS

Blood was taken from mice 48 hours following their ninth dose of quinacrine (day 19 of the toxicity study) via saphenous vein bleed. Blood was then centrifuged at 500 × g to separate plasma from cells. Plasma levels of quinacrine were quantified using ultra-high performance liquid chromatography (HPLC) combined with mass spectroscopy (MS) (Supplementary Figure 2). For detailed methods, see Supplementary Appendix.

### Visualizing drug penetration of tumors

To establish whether quinacrine was able to penetrate into tumor tissue, fresh frozen tumors were sectioned using a cryostat (Bright Instruments, Huntingdon, UK) and mounted using anti-fade mountant (Thermo Fisher Scientific, MA, USA) containing DAPI (4′,6-diamidino-2-phenylindole). The fluorescence caused by quinacrine was then imaged at 488 nm using an epifluorescence microscope.

### Statistics

Data were represented as mean ± standard error of the mean (SEM) unless otherwise described. One-Way ANOVA followed by Dunnett’s or Tukey’s corrections for multiple comparisons was used to analyze all data other than for clonogenic analysis, which utilized Two-Way ANOVA. Each treatment value was compared to that of the appropriate control as indicated in the figures. Relative changes were calculated by dividing the value of the drug-treated sample by that of the untreated sample. Kaplan-Meier analyses were used to show differences in survival between all groups. A Mantel-Haenszel test was carried out to calculate the hazard ratios of death for the different treatments on tumor-bearing mice. All statistical analysis was completed using GraphPad Prism 6.

## SUPPLEMENTARY MATERIALS


