# Supplementary material for: Repurposed quinacrine synergizes with cisplatin, reducing the effective dose required for treatment of head and neck squamous cell carcinoma
Source: Oncotarget. 2019 Aug 27;10(50):5229–44. doi: 10.18632/oncotarget.27156 (PMC6718257; doi:10.18632/oncotarget.27156)
Supplement: Supplementary file 1 [file oncotarget-10-5229-s001.pdf]

Repurposed quinacrine synergizes with cisplatin, reducing the effective dose required for treatment of head and neck squamous cell carcinoma

SUPPLEMENTARY MATERIALS

MATERIALS AND METHODS

Quantification of quinacrine in the blood using ultra-high performance liquid chromatography-mass spectrometry (UHPLC-MS)

Drug standard preparation

Quinacrine stock solution was made up in water (LC-MS grade, VWR). An isotopically labelled internal standard of quinacrine was not available.

Sample preparation

Plasma samples from quinacrine treated mice were extracted to retain metabolites and drugs, while removing proteins, RNA and DNA as follows: ice-cold acetonitrile (LC-MS grade, VWR) was added to plasma (3:1 acetonitrile:plasma) and samples were vortexed (30 s) and centrifuged (13 000 ×g, 4°C, 20 min). Supernatant was loaded into a clean HPLC vial for UHPLC-MS analysis. Standard curve samples were prepared by spiking quinacrine into plasma from untreated mice (drug range 3.2 ng/mL to 5 µg/mL- initially six points plus a blank) and extracting the plasma with acetonitrile as above.

Ultra-high performance liquid chromatography-mass spectrometry (UHPLC-MS)

All samples were acquired in duplicate using a Dionex UltiMate 3000 Rapid Separation LC system (Thermo Fisher Scientific, MA, USA) coupled with a heated electrospray Q Exactive Focus mass spectrometer (Thermo Fisher Scientific, MA, USA). Extracts were analysed on a Hypersil GOLD column (100 × 2.1 mm, 1.9 µm; Thermo Fisher Scientific, MA, USA). Mobile phase A consisted of water with 0.1% formic acid and mobile phase B consisted of acetonitrile with 0.1% formic acid. The flow rate was 0.40 mL/min with the following gradient: t = 0 min, 1% B; t = 1 min, 1% B; t = 8 min, 99% B; t = 12 min, 99% B; t = 13 min, 1% B; t = 16 min = end [all changes were linear with curve = 5]. The column temperature was set to 45°C and the injection volume was 2 µL. Data were acquired in positive ionisation mode within the mass range of 350 – 450 m/z at resolution 35 000 (FWHM at m/z 200). To confirm the identity of quinacrine peaks in each sample a HCD MS/MS event was triggered on the accurate mass of the $[M+H]^+$ quinacrine ion (400.21502 m/z). HCD MS/MS settings were: isolation width = 3 m/z, 17 500 resolution and stepped normalised collision energies (stepped NCE) of 20, 50, 80%. Ion source parameters were set as follows: Sheath gas = 30 arbitrary units, Aux gas = 12 arbitrary units, Sweep gas = 0 arbitrary units, Spray Voltage = 3.2kV, Capillary temp. = 380°C, Aux gas heater temp. = 350°C.

Quantification of quinacrine in the blood using ultra-high performance liquid chromatography-mass spectrometry (UHPLC-MS)

The identity of the $[M+H]^+$ quinacrine ion in plasma was confirmed by comparing the its MS/MS spectrum to that of the standard (Supplementary Figure 2). The final quinacrine standard curve contained 4 points (each in duplicate) plus a zero point (2 original points were removed as they fell outside of the linear range). The standard curve had a lower limit of quantification of 16 ng/mL and an upper limit of quantification of 2 µg/mL. The standard curve R^2 coefficient was 0.9979 and the accuracy for all data points (relative error) was within ±17%.

Data analysis

To confirm the identity of quinacrine in samples, the HCD MS/MS spectrum and retention time of the $[M+H]^+$ quinacrine ion (400.21502 m/z) was visually compared with that from a quinacrine standard. Data was processed using Quan Browser software (Thermo Fisher Scientific, MA, USA). Chromatographic peaks corresponding to the $[M+H]^+$ quinacrine ion were integrated for all samples and standard samples. A standard curve was plotted in Excel, and the equation of the linear curve was used to quantify quinacrine chromatographic peaks in samples.

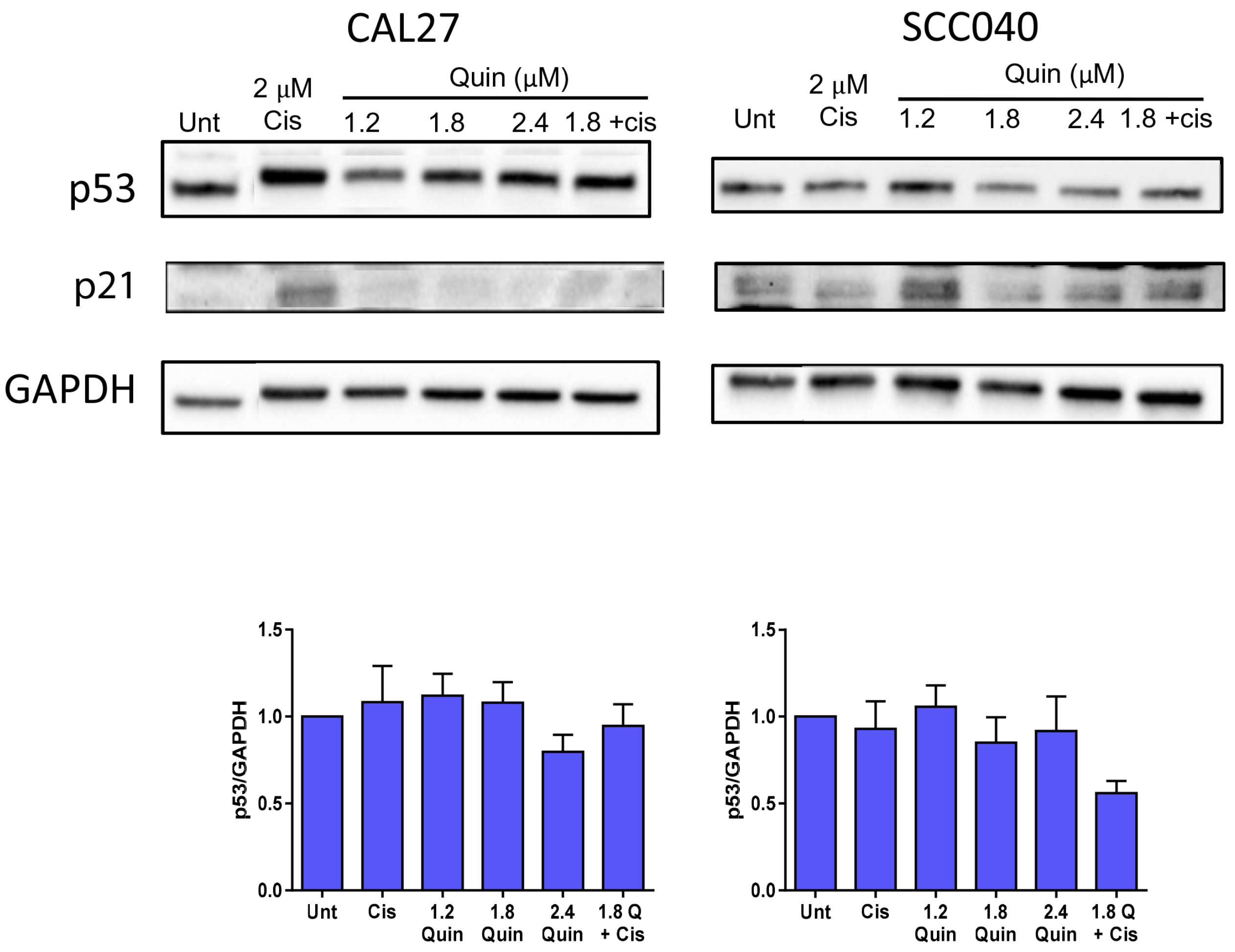

Supplementary Figure 1: Western blot analysis of p53 (*n* = 3) and p21 (*n* = 2) protein expression in CAL27 and SCC040 following 48 hours exposure to quinacrine alone (1.2, 1.8 and 2.4 μM), 1.8 μM quinacrine + 2 μM cisplatin and 2 μM cisplatin alone. GAPDH was used as a loading control. No differences in expression compared with untreated controls were observed.

A

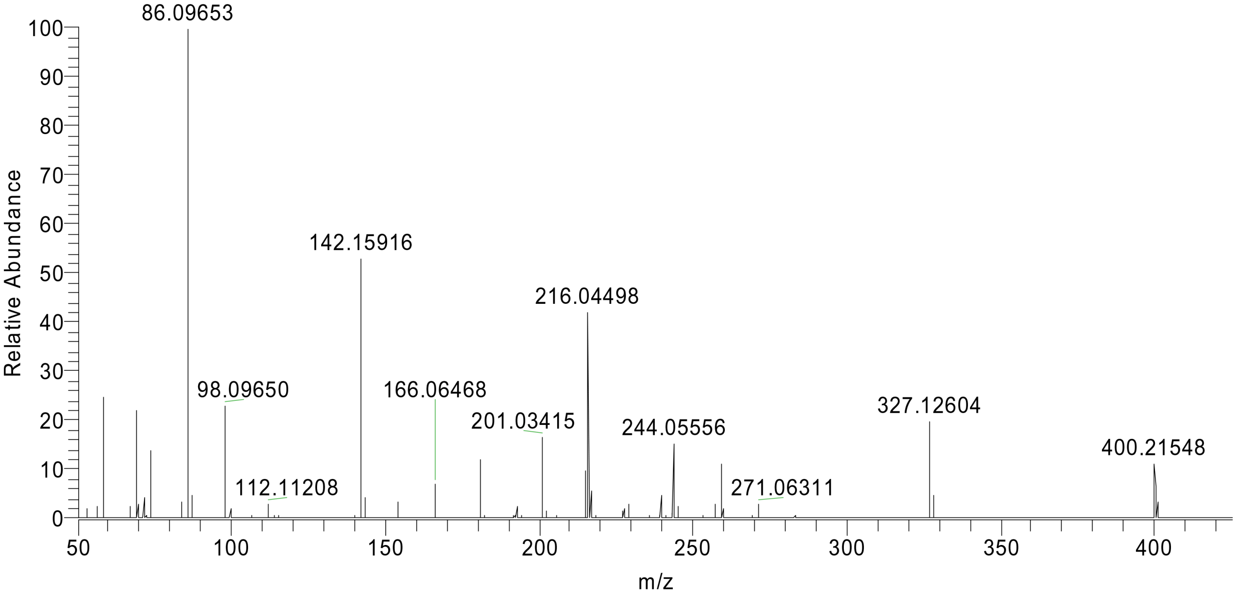

B

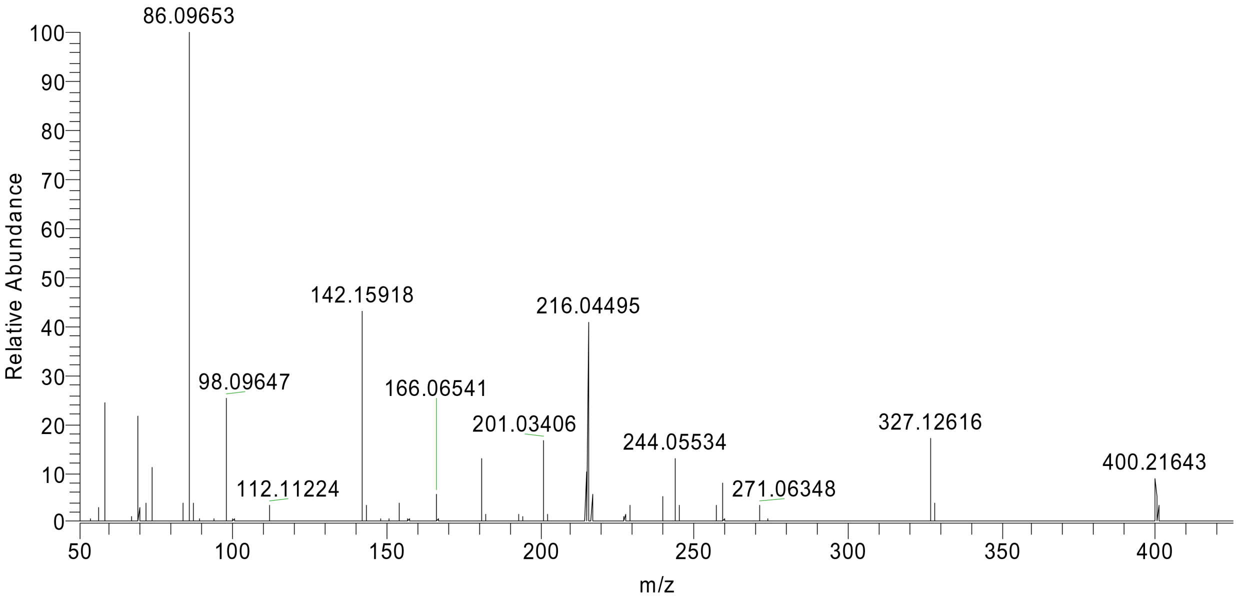

Supplementary Figure 2: Mass spectra showing higher-energy C-trap dissociation (HCD) MS/MS fragmentation analysis of the $[M+H]^+$ quinacrine ion in. (**A**) the chemical standard and (**B**) a plasma sample from a mouse treated with 100 mg/kg quinacrine 48 hours prior to having blood samples taken for analysis.
